# From Synergy to Strain: Exploring the Psychological Mechanisms Linking Employee–AI Collaboration and Knowledge Hiding

**DOI:** 10.3390/bs16010013

**Published:** 2025-12-20

**Authors:** Yi-Bin Li, Ting-Hsiu Liao, Chih-Hao Tsai, Tung-Ju Wu

**Affiliations:** 1 Business School, Huaqiao University, Quanzhou 362021, China; liyibin@hqu.edu.cn; 2 Department of Tourism and Recreation, Cheng Shiu University, Kaohsiung 83347, Taiwan; 8481@gcloud.csu.edu.tw; 3 Department of Hospitality and MICE Marketing Management, National Kaohsiung University of Hospitality and Tourism, Kaohsiung 81271, Taiwan; chtsai@mail.nkuht.edu.tw; 4 School of Management, Harbin Institute of Technology, Harbin 150001, China

**Keywords:** job insecurity, AI trust, employee–AI collaboration, Cognitive Appraisal Theory, knowledge hiding

## Abstract

As artificial intelligence (AI) becomes an integral part of organizational operations, collaboration between humans and AI is transforming employees’ work experiences and behavioral patterns. This study examines the psychological challenges and coping responses associated with such collaboration. Drawing on Cognitive Appraisal Theory, we construct and test a theoretical framework that connects employee–AI collaboration to knowledge hiding via job insecurity, while considering AI trust as a moderating variable. Data were collected through a three-wave time-lagged survey of 348 employees working in knowledge-intensive enterprises in China. The empirical results demonstrate that (1) employee–AI collaboration elevates perceptions of job insecurity; (2) job insecurity fosters knowledge-hiding behavior; (3) job insecurity mediates the link between collaboration and knowledge hiding; and (4) AI trust buffers the positive effect of collaboration on job insecurity, thereby reducing its indirect impact on knowledge hiding. These findings reveal the paradoxical role of AI collaboration: although it enhances efficiency, it may also provoke defensive reactions that inhibit knowledge exchange. By highlighting the role of AI trust in shaping employees’ cognitive appraisals, this study advances understanding of how cognitive appraisals influence human adaptation to intelligent technologies. Practical insights are offered for managers aiming to cultivate trust-based and psychologically secure environments that promote effective human–AI collaboration and organizational innovation.

## 1. Introduction

With the accelerated advancement of artificial intelligence (AI), a growing number of organizations have begun embedding AI technologies into both their operational processes and strategic decision-making (Li et al., 2023; Wu et al., 2025a). In addition to its extensive utilization across sectors such as manufacturing, finance, healthcare, and education, AI has progressively penetrated knowledge-intensive areas including human resource management, innovation and research and development, and customer relationship management (Budhwar et al., 2023). Although AI adoption has proven highly effective in improving efficiency, cutting costs, and enhancing the quality of decision-making, its deeper influence lies in transforming the very nature of work and the architecture of organizational systems (Li et al., 2025a; Perez et al., 2024). Against this backdrop, employee–AI collaboration has become a new organizational normal: human employees and AI complement one another’s strengths and jointly undertake complex tasks, thereby generating synergistic effects in data processing, knowledge integration, and problem solving that surpass the capabilities of either party alone (Kong et al., 2023). Thus, employee–AI collaboration is not only an inevitable consequence of technological advancement but also a critical avenue for organizations to sustain their competitive advantage. However, the diffusion of employee–AI collaboration also introduces a novel work context that requires employees to actively interpret and evaluate how working alongside AI affects their roles, value, and future prospects (B. J. Kim & Lee, 2025). While such collaboration reconfigures workflows, it also reshapes employees’ psychological experiences, raising important questions about how employees cognitively appraise AI-enabled collaboration and how these appraisals influence their subsequent behaviors.

In examining the organizational outcomes of employee–AI collaboration, the phenomenon of knowledge hiding warrants particular attention (Connelly et al., 2012; Wu et al., 2025b). Knowledge constitutes a vital resource for organizational survival and growth, and knowledge sharing has long been regarded as the cornerstone of innovation and sustained competitive advantage. Yet, extensive research has shown that employees often withhold knowledge during interactions. Specifically, when confronted with requests for knowledge, they may deliberately conceal or partially conceal information—a behavior known as knowledge hiding (Anand et al., 2022). Unlike simple knowledge loss or forgetfulness, knowledge hiding is intentional and motivated, making its detrimental effects on teamwork and organizational performance especially severe (Connelly et al., 2019). In the context of employee–AI collaboration, this issue may become even more salient. The rapid learning and substitution capabilities of AI may lead employees to fear that their knowledge advantages are no longer unique or that their future positions may be jeopardized. Consequently, they may become more inclined to retain, rather than share, knowledge (Nguyen et al., 2022). Such behaviors not only erode trust and cooperation within teams but also hinder organizations from fully leveraging AI to integrate knowledge and foster innovation (Černe et al., 2014). 

Although research on AI-driven transformations and knowledge hiding has grown rapidly, existing studies primarily focus on technological turbulence, job displacement risk, or generalized threat perceptions associated with AI, rather than examining how employees cognitively interpret and evaluate employee–AI collaboration as an ongoing work relationship. Prior research has largely treated AI as a contextual force that triggers negative reactions, without sufficiently unpacking the cognitive appraisal processes through which employees make sense of collaboration with AI and translate such interpretations into specific coping-oriented behaviors, such as knowledge hiding. In particular, little is known about how employee–AI collaboration is appraised as a potential threat to job continuity and role value, or how individual differences—such as trust in AI—shape these appraisals and their behavioral consequences. For example, Arias-Pérez and Vélez-Jaramillo (2022) show that AI-induced technological turbulence increases knowledge hiding through heightened AI threat awareness; G. Xu and Xue (2023) reveal that unemployment risk perception triggers knowledge hiding via psychological contract breach; B. J. Kim and Kim (2024) demonstrate that AI-induced job insecurity reduces psychological safety, which in turn promotes knowledge hiding, while AI learning self-efficacy mitigates this effect; Chen et al. (2025) distinguish between instrumental and emotional AI interaction and find that instrumental AI use heightens job insecurity and knowledge hiding; similarly, C. Xu et al. (2024) confirm that AI application elevates job insecurity, thereby fostering unethical behaviors such as knowledge hiding. Although these studies collectively emphasize the defensive responses triggered by AI-related threats, they tend to focus on AI introduction, AI usage patterns, or perceived replacement potential, rather than examining how employee–AI collaboration as an ongoing working relationship reshapes employees’ threat-based interpretations of their roles and future prospects and influences their knowledge behaviors. Moreover, prior research largely examines antecedents such as AI threat, AI awareness, interaction type, or perceived substitution, but rarely conceptualizes collaboration itself as a contextual force capable of altering employees’ role identity, threat-based appraisals, and behavioral strategies. Likewise, although scholars have begun to acknowledge the role of individual attitudes—such as AI self-efficacy or perceived support—few studies have considered trust in AI as a cognitive appraisals that may buffer insecurity during collaboration. Given that AI trust influences whether employees perceive AI as a supportive partner or a threatening competitor, its moderating role in the link between employee–AI collaboration and knowledge hiding remains insufficiently explored. To address these research gaps, the research question of this study is: Does employee–AI collaboration induce knowledge hiding by heightening employees’ threat-based perceptions of job insecurity, and does AI trust mitigate both the insecurity triggered by collaboration and its indirect impact on knowledge hiding?

From a theoretical perspective, Cognitive Appraisal Theory provides a valuable framework for understanding employees’ psychological and behavioral responses to employee–AI collaboration (Lazarus & Folkman, 2006). This theory posits that individuals do not respond to environmental changes directly; rather, they first engage in cognitive evaluations to determine whether a given situation poses a threat or an opportunity (Lazarus & Folkman, 2006). Applied to employee–AI collaboration, working alongside AI can be cognitively appraised as a threat to job continuity, role relevance, or future career prospects, thereby eliciting job insecurity as a threat-based appraisal (Cao & Song, 2025). In response to such appraisals, employees may adopt defensive coping behaviors—such as knowledge hiding—to protect themselves from perceived negative consequences (Gibbard et al., 2025; Hellgren et al., 1999). Moreover, employees’ trust in AI shapes how collaboration is cognitively interpreted, influencing whether it is appraised as threatening or benign (De Witte et al., 2016). By adopting this perspective, the present study examines how employee–AI collaboration triggers job insecurity through cognitive appraisal processes and how such appraisals translate into knowledge-hiding behaviors.

However, the psychological and behavioral consequences of employee–AI collaboration are not uniform or static; instead, they vary depending on individual characteristics and contextual conditions. Among these influencing factors, trust in AI emerges as a crucial determinant (Kong et al., 2023). AI trust reflects the degree to which employees perceive AI as competent, dependable, and consistent with their personal or professional objectives (Ueno et al., 2022). Employees with strong trust in AI tend to regard it as a collaborative ally that enhances their capabilities and facilitates work outcomes rather than as a potential threat (Lu et al., 2025). Such trust can mitigate the job insecurity induced by employee–AI collaboration and, in turn, reduce knowledge hiding. Conversely, when employees lack trust in AI, they are more inclined to interpret its involvement as a potential threat, amplifying insecurity and defensive reactions, thereby increasing the likelihood of knowledge hiding (Pan et al., 2018). Accordingly, AI trust is not only a psychological condition for the smooth advancement of employee–AI collaboration but also a crucial boundary variable shaping its relationship with knowledge hiding.

In summary, this study builds on Cognitive Appraisal Theory to propose a theoretical model that integrates both mediating and moderating mechanisms. Specifically, employee–AI collaboration fosters knowledge hiding by intensifying employees’ job insecurity, whereas AI trust moderates this process by alleviating insecurity and weakening its downstream effects. Through the construction and empirical examination of this model, the present research enhances understanding of knowledge-hiding behaviors within the context of employee–AI collaboration and broadens the applicability of Cognitive Appraisal Theory to emerging technological environments. Furthermore, the findings provide actionable insights for organizational practice: when introducing and promoting AI, managers should not only prioritize efficiency gains and technological integration but also pay close attention to employees’ psychological experiences and trust-building, thereby reducing knowledge hiding and fostering knowledge sharing and innovation in the AI era.

## 2. Theory and Hypotheses

### 2.1. Theoretical Background

Cognitive Appraisal Theory represents a foundational framework for explaining how individuals psychologically and behaviorally respond to environmental changes and work-related stressors (Lazarus & Folkman, 2006). Rather than reacting automatically to external stimuli, individuals first engage in cognitive evaluations to assess whether a given situation poses a threat, a challenge, or is benign. These appraisals shape subsequent emotional reactions and behavioral responses. According to this perspective, stress and strain do not arise directly from objective conditions but from individuals’ subjective interpretations of how those conditions affect their goals, roles, and future prospects.

Applied to contemporary work contexts, Cognitive Appraisal Theory is particularly relevant for understanding employees’ reactions to employee–AI collaboration. Working alongside AI introduces a novel and dynamic work situation that may be cognitively evaluated as threatening employees’ job continuity, role relevance, or long-term career prospects. When such collaboration is appraised as a threat, employees are likely to experience job insecurity, which reflects a subjective perception of uncertainty and concern regarding the stability and value of one’s job (De Witte et al., 2016; Lee et al., 2018). In this sense, job insecurity can be understood as a threat-based cognitive appraisal that emerges when employees interpret employee–AI collaboration as potentially harmful to their occupational future.

Cognitive Appraisal Theory further posits that individuals respond to threat-based appraisals through coping behaviors aimed at managing or mitigating perceived negative consequences (Lazarus & Folkman, 2006). Such coping responses may be emotional or behavioral in nature and often involve defensive strategies designed to protect the self. In knowledge-intensive work settings, one salient coping behavior is knowledge hiding—employees’ deliberate concealment of requested knowledge. When employees cognitively appraise employee–AI collaboration as threatening, knowledge hiding may serve as a defensive coping response intended to preserve perceived control, value, or indispensability within the organization (Connelly et al., 2012; Serenko & Bontis, 2016).

Importantly, Cognitive Appraisal Theory emphasizes that cognitive evaluations are not uniform across individuals but are shaped by personal beliefs and contextual cues. In the context of employee–AI collaboration, trust in AI plays a critical role in shaping how employees interpret collaboration with intelligent systems. Employees with higher levels of AI trust are more likely to appraise collaboration as supportive or opportunity-enhancing, whereas those with lower trust are more inclined to interpret it as threatening. Consequently, AI trust functions as a key boundary condition that influences whether employee–AI collaboration is cognitively appraised as a threat and how such appraisals translate into subsequent coping behaviors. By adopting Cognitive Appraisal Theory as the core theoretical lens, the present study explains how employee–AI collaboration triggers job insecurity through cognitive appraisal processes and how these appraisals shape knowledge-hiding behaviors.

### 2.2. Employee–AI Collaboration and Job Insecurity

Employee–AI collaboration refers to a complementary working relationship between human employees and AI during task execution (Kong et al., 2023). Unlike simple tool usage, employee–AI collaboration emphasizes interaction and division of labor between humans and machines in cognition, decision-making, and action: AI excels at rapid computation, pattern recognition, and information processing, whereas humans hold advantages in complex judgment, contextual understanding, and emotional communication (Li et al., 2024). As AI becomes increasingly embedded in organizational processes, employee–AI collaboration has evolved into a critical component of workflows, affecting not only efficiency but also employees’ role identity and psychological experience (Li et al., 2025a, 2025b).

Job insecurity refers to employees’ perceptions of uncertainty and worry regarding the stability and continuity of their employment (Lee et al., 2018). This construct encompasses both direct worries about job loss and indirect perceptions of diminished job roles or value (Sverke et al., 2006). Prior studies have shown that job insecurity is a prototypical stressor that undermines job satisfaction and organizational commitment and elicits negative behavioral responses (Cheng & Chan, 2008; László et al., 2010). In particular, in contexts of rapid technological change, such insecurity becomes more salient as employees fear that their competencies and value may be replaced by new technologies (Jiang & Lavaysse, 2018).

Moreover, employee–AI collaboration represents an inherently ambivalent work context that requires continuous cognitive evaluation by employees. While collaboration with AI may create opportunities for learning and skill development, it simultaneously prompts employees to compare their own capabilities with those of AI during joint task execution. Such ongoing comparisons heighten employees’ attention to cues related to their role relevance and future employability. When organizations fail to provide sufficient training, guidance, or clarity regarding the division of labor between humans and AI, employees are more likely to interpret collaboration as threatening rather than opportunity-enhancing (Li et al., 2023). Prior research has shown that rapid advances in intelligent technologies intensify employees’ concerns about potential substitution and career uncertainty (Koo et al., 2021; Wu et al., 2024; Tu et al., 2023; Sharif et al., 2025). From a cognitive appraisal perspective, employee–AI collaboration therefore constitutes a salient work condition that may be appraised as a threat to job continuity, skill value, and long-term career prospects, thereby increasing employees’ job insecurity.

Furthermore, employee–AI collaboration differs from traditional forms of technological change in that it involves sustained, task-level interaction with intelligent systems, making employees’ threat appraisals more immediate and psychologically salient. Rather than responding to AI adoption as a distant organizational decision, employees experience collaboration with AI as an ongoing work condition that directly shapes how they interpret their own value and replaceability. As AI systems increasingly demonstrate advanced learning and problem-solving capabilities, employees may interpret collaboration as a signal that their expertise is becoming less distinctive or less indispensable (Koo et al., 2021). Such interpretations reinforce concerns about job replacement, reduced promotion opportunities, and constrained career development paths (Wu et al., 2024; Tu et al., 2023; Sharif et al., 2025). When employees cognitively evaluate these signals as threatening to their occupational future, they are more likely to experience heightened job insecurity, defined as a subjective perception of uncertainty regarding job continuity and role stability (Lee et al., 2018). Accordingly, employee–AI collaboration is expected to positively influence employees’ job insecurity. Thus, we hypothesize:

**Hypothesis** **1.** *Employee–AI collaboration is positively influences job insecurity.*

### 2.3. Job Insecurity and Knowledge Hiding

Job insecurity reflects employees’ concerns about job continuity and role stability, encompassing both direct fears of job loss and indirect perceptions of diminished career opportunities and skill value (B. J. Kim & Lee, 2025). Extensive research demonstrates that job insecurity is a stressor that provokes anxiety, tension, and negative emotions, thereby influencing work attitudes and behaviors (Ibrahim Hassan et al., 2024; Serenko & Bontis, 2016). Knowledge hiding refers to employees’ deliberate concealment or partial concealment of requested knowledge. Unlike unintentional knowledge withholding or forgetting, knowledge hiding is intentional and motivated, often manifested as pretending not to know, redirecting conversations, or giving excuses for non-disclosure (Fauzi, 2023). This behavior undermines trust within teams, obstructs knowledge flow, and impedes innovation, thereby harming organizational performance (Khoreva & Wechtler, 2020).

Based on a cognitive appraisal perspective, a strong link can be established between job insecurity and knowledge hiding. When employees cognitively appraise their work situation as threatening to job continuity or future career prospects, they are more likely to adopt defensive coping strategies aimed at reducing perceived vulnerability. In knowledge-intensive contexts, knowledge represents a critical means through which employees signal competence, maintain distinctiveness, and safeguard their position within the organization. Accordingly, employees who experience heightened job insecurity may deliberately conceal requested knowledge as a way of coping with perceived threats and preserving their sense of control and irreplaceability (Rezwan & Takahashi, 2021; Zhao et al., 2016). This defensive coping logic becomes particularly salient in contexts characterized by rapid technological change. When employees perceive uncertainty regarding their future roles or skill relevance, they are less inclined to engage in cooperative behaviors and more likely to prioritize self-protective actions (Jiang & Lavaysse, 2018). In the context of employee–AI collaboration, these tendencies may be further amplified, as employees may interpret the presence of AI as increasing the risk that shared knowledge could be replicated or rendered less valuable. Such threat-based interpretations strengthen employees’ motivation to withhold knowledge as a coping response to perceived job insecurity (Y. Zhang et al., 2023).

Prior studies also indicate that job insecurity undermines organizational commitment and willingness to collaborate, leading employees to favor self-protective rather than organizationally oriented behaviors (Arain et al., 2020). Specifically, concerns about uncertain career trajectories prompt employees to limit knowledge contributions in teams to avoid exploitation or marginalization (Oliveira et al., 2021). Building on this foundation, the present study extends prior research by situating these dynamics within the emerging context of human–AI collaboration, a setting in which perceived threats may be heightened due to AI’s learning and substitutive capabilities. Accordingly, job insecurity may act as a critical antecedent to knowledge hiding, particularly when employees feel that collaboration with AI intensifies uncertainties regarding their value and long-term employability.

**Hypothesis** **2.** *Job insecurity positively influences knowledge hiding.*

### 2.4. The Mediating Role of Job Insecurity

The above discussion has demonstrated that employee–AI collaboration is associated with employees’ perceptions of job insecurity, which in turn are linked to knowledge-hiding behaviors. Based on these established relationships, this study posits that job insecurity functions as a mediating mechanism between employee–AI collaboration and knowledge hiding. First, although employee–AI collaboration presents new opportunities, it simultaneously introduces psychological strain by altering how employees interpret their roles and future prospects (Li et al., 2023). While AI may improve efficiency and alleviate workloads, it also raises concerns about substitution and skill devaluation (Li et al., 2025a). When working alongside AI, employees may become increasingly aware that their expertise appears less distinctive or may fear eventual replacement (L.-X. Zhang et al., 2023). Such interpretations give rise to job insecurity, reflecting threat-based appraisals regarding job continuity, role relevance, and career development.

Subsequently, job insecurity motivates defensive coping behaviors aimed at managing perceived threats and reducing vulnerability (Sharif et al., 2025). In knowledge-intensive contexts, knowledge represents a critical means through which employees signal competence and maintain perceived indispensability. Employees experiencing heightened insecurity are therefore more likely to withhold knowledge as a way of preserving control over their work value, even when such behavior undermines cooperation and performance (Jiang & Lavaysse, 2018; Y. Zhang et al., 2023). In the context of employee–AI collaboration, this tendency may be further amplified because employees not only worry about job instability but also interpret AI’s capacity to learn and replicate knowledge as intensifying threats to their professional distinctiveness. As a result, employees may become increasingly inclined to engage in knowledge hiding as a defensive coping strategy.

Finally, from a dynamic perspective, insecurity arising from employee–AI collaboration may persist and intensify over time rather than dissipate immediately (B. J. Kim & Lee, 2025). As AI becomes more deeply embedded in daily work processes, employees’ concerns about role relevance and job stability may become more salient, exerting sustained influence on knowledge-sharing behaviors (Li et al., 2023). Prolonged job insecurity fosters a heightened threat-oriented interpretive state in which employees increasingly rely on defensive coping responses, such as knowledge hiding, to manage ongoing uncertainty and perceived risks. By conceptualizing employee–AI collaboration as a work context that elicits threat-based appraisals and defensive behavioral responses, this study extends existing knowledge-hiding research into the domain of human–AI collaboration. Thus, we hypothesize:

**Hypothesis** **3.** *Job insecurity mediates the influences between employee–AI collaboration and knowledge hiding.*

### 2.5. The Moderating Role of AI Trust

AI trust is defined as employees’ belief in the competence, reliability, and value congruence of AI (Glikson & Woolley, 2020). It reflects employees’ subjective judgments regarding whether AI can reliably execute tasks and support their work goals (Choung et al., 2023). Beyond being a technological perception, AI trust represents a cognitive belief system that shapes how employees interpret and make sense of their interactions with AI, thereby influencing their attitudes and behavioral responses (Habbal et al., 2024). As noted earlier, although employee–AI collaboration can enhance efficiency and innovation, it may simultaneously evoke job insecurity. However, employees do not interpret collaboration with AI uniformly; rather, their interpretations are shaped by their level of trust in AI (Omrani et al., 2022).

When employees have high trust in AI, they are more likely to cognitively interpret collaboration as supportive and complementary rather than threatening. High AI trust encourages employees to view AI as a collaborative partner that enhances human performance and compensates for human limitations, instead of as a substitute that undermines human roles (Ferrario & Loi, 2022). As a result, employee–AI collaboration is less likely to be cognitively appraised as a signal of diminished skill value or reduced job relevance. Such benign interpretations reduce employees’ concerns about devaluation and career uncertainty, thereby weakening the positive relationship between employee–AI collaboration and job insecurity (de Brito Duarte et al., 2023).

Conversely, when employees exhibit low trust in AI, they tend to approach collaboration with greater suspicion and defensiveness, interpreting AI adoption as an indication of instability and constrained personal development (Gillath et al., 2021). Under these conditions, employees are more inclined to cognitively appraise employee–AI collaboration as a threat to their roles and future prospects, viewing AI as a potential competitor capable of replacing or marginalizing human contributions. Low AI trust intensifies threat-based interpretations of collaboration and amplifies employees’ concerns about job continuity and role relevance, thereby strengthening job insecurity triggered by employee–AI collaboration (Bedué & Fritzsche, 2022). Accordingly, differences in AI trust fundamentally shape whether employee–AI collaboration is cognitively appraised as relatively benign or threatening.

Based on this reasoning, AI trust should serve as a critical boundary condition that determines how employee–AI collaboration is cognitively appraised and, consequently, the strength of its relationship with job insecurity. Accordingly, we hypothesize:

**Hypothesis** **4.** *AI trust moderates the influences on employee–AI collaboration and job insecurity such that the positive influences is weaker when AI trust is high and stronger when AI trust is low.*

Moreover, drawing on Cognitive Appraisal Theory, we contend that AI trust moderates the indirect relationship between employee–AI collaboration and knowledge hiding through job insecurity by shaping employees’ cognitive interpretations of collaboration with AI. Cognitive Appraisal Theory suggests that individuals’ behavioral responses to stressful situations depend not only on the presence of external stimuli but also on how these situations are cognitively evaluated as threatening or non-threatening. In this context, AI trust influences whether employee–AI collaboration is cognitively appraised as a potential threat to job continuity and role value or as a benign and manageable work condition. When employees exhibit high levels of AI trust, they are more likely to interpret collaboration with AI in a non-threatening manner, which reduces threat-based appraisals of job insecurity and, in turn, weakens the motivational basis for defensive coping behaviors such as knowledge hiding (J. Kim et al., 2021; Kaplan et al., 2023; Mylrea & Robinson, 2023). High AI trust therefore attenuates the extent to which job insecurity mediates the relationship between employee–AI collaboration and knowledge hiding by mitigating employees’ threat-oriented interpretations.

In contrast, when AI trust is low, employees are less confident in AI’s reliability, intentions, and implications for human roles, making them more likely to cognitively appraise employee–AI collaboration as a direct threat to job stability, skill relevance, and future career prospects. Such threat-based appraisals intensify feelings of job insecurity and reinforce defensive coping tendencies aimed at self-protection, including knowledge hiding (Asan et al., 2020; Gerlich, 2024; Reinhardt, 2023). Under conditions of low AI trust, threat interpretations become more salient, strengthening the psychological mechanism through which job insecurity translates into knowledge-hiding behavior. Consequently, AI trust not only moderates the direct relationship between employee–AI collaboration and job insecurity but also conditions the strength of the downstream indirect effect of collaboration on knowledge hiding via job insecurity. Thus, we propose:

**Hypothesis** **5.** *AI trust moderates the indirect effect of employee–AI collaboration on knowledge hiding through job insecurity such that the indirect effect is weaker when AI trust is high and stronger when AI trust is low.*

**Figure 1:**
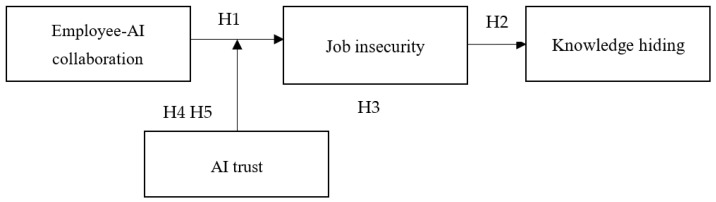
Research Model.

In summary, the proposed research model is depicted in Figure 1.

## 3. Method

### 3.1. Procedure and Sample

In terms of sampling strategy, this study adopted a non-random, convenience sampling method. Participating enterprises were recruited through existing professional contacts and voluntary cooperation from HR departments, and employees self-selected into the study. Because access to organizations that have implemented AI applications requires prior authorization and coordination, a random sampling procedure was not feasible. Convenience sampling is widely used in organizational behavior research, particularly when examining emerging technological contexts such as AI-enabled workplaces. Despite being non-random, the sample reflects a diverse range of knowledge-intensive industries—including information technology, financial services, and manufacturing—and thus provides a reasonable level of representativeness for employees working in AI-supported environments. 

To strengthen causal inference and minimize potential common method bias, this research adopted a three-wave time-lagged survey approach. Unlike a single cross-sectional design, the three-phase data collection enabled temporal separation among the independent, mediating, and dependent variables, thereby mitigating same-source bias and offering more reliable evidence for the proposed causal mechanisms. Specifically, the participating enterprises were primarily located in Fujian Province, which has actively promoted digital transformation and AI adoption in recent years. This regional focus provides a representative context for examining employee–AI collaboration in knowledge-intensive industries.

The data collection procedure was as follows. At Time 1 (T1, June 1, 2025), during the project initiation phase, the research team collaborated with HR departments to introduce the purpose and confidentiality principles of the study and obtained voluntary participation from employees. At this stage, employees reported their perceptions of employee–AI collaboration, their level of AI trust, and demographic information (e.g., gender, age, education, tenure). These measures captured employees’ initial subjective experiences of collaboration with AI. At Time 2 (T2, June 15, 2025), one week after T1, the same participants completed a second survey focusing on job insecurity. The time lag was intended to allow employees to further experience employee–AI collaboration in their daily work, thereby eliciting more accurate perceptions of job stability and role value. At Time 3 (T3, June 30, 2025), approximately one week after T2, the participants completed the third survey, which measured knowledge hiding. By measuring the dependent variable at the final wave, we reduced the likelihood of same-source bias stemming from simultaneous assessment of predictors and outcomes. To ensure accurate matching of responses across waves, each participant created an anonymous code and retained it for use in all three surveys.

Data were collected from knowledge-intensive enterprises located in eastern and central China, including firms in information technology, financial services, and manufacturing. These organizations had adopted AI tools or systems in their daily operations, providing typical contexts for employee–AI collaboration. With the support of HR departments, surveys were distributed both online and offline. At T1, 500 questionnaires were distributed and 462 returned; after excluding invalid responses (e.g., excessive missing data, straight-line responses), 440 valid responses remained. At T2, surveys were sent to these participants, yielding 402 responses, of which 389 were valid. At T3, 360 responses were received, of which 348 were valid. After matching across all three waves, a final sample of 348 valid participants was obtained, representing an overall effective response rate of 69.6%.

Among the 348 respondents, 56.3% were male and 43.7% female. The average age was 32.4 years (*SD* = 6.8), with the majority aged between 25 and 40. Average tenure was 7.2 years (*SD* = 5.1). In terms of industry distribution, 40.5% worked in information technology, 33.6% in financial services, and 25.9% in manufacturing. The sample characteristics align well with the knowledge-intensive occupational context of the study, ensuring representativeness.

### 3.2. Measures

All key constructs were assessed using measurement scales that have been validated in previous studies. Prior to administering the main questionnaire, a pilot test involving a small sample was carried out to ensure the reliability and suitability of the measurement instruments. With the exception of demographic variables, all items were rated on a five-point Likert scale ranging from 1 (strongly disagree) to 5 (strongly agree).

*Employee–AI Collaboration* Measured using the human–machine interaction scale developed by Kong et al. (2023), which assesses employees’ perceptions of the extent to which AI collaborate with them in daily tasks. Data were collected at T1. Cronbach’s α = 0.92.

*AI Trust* Measured using the scale by McGrath et al. (2025), which captures employees’ trust in the competence, reliability, and goal alignment of AI. Data were collected at T1. Cronbach’s α = 0.88.

*Job Insecurity* Measured using the classical scale developed by Hellgren et al. (1999), which has been widely validated in subsequent research. Data were collected at T2. Cronbach’s α = 0.82.

*Knowledge Hiding* Measured using the scale developed by Connelly et al. (2012). Employees were instructed to respond based on their work experiences over the past month. Data were collected at T3. Cronbach’s α = 0.84.

*Control Variables* To rule out potential confounding effects, we controlled for gender and age, both of which have been shown in prior research to influence knowledge behaviors and job insecurity.

## 4. Results

### 4.1. Test for Common Method Bias

To examine the possibility of common method bias, Harman’s single-factor test was first performed. All measurement items were subjected to an unrotated exploratory factor analysis. The analysis revealed that the first factor accounted for 22.76% of the total variance, with an eigenvalue exceeding 1—substantially lower than the commonly accepted 40% threshold. This outcome indicates that common method bias is unlikely to pose a significant problem in the present research.

Recognizing that Harman’s approach may not fully capture potential bias, an additional test was conducted by incorporating a latent error factor into the five-factor measurement model. Comparison between this modified model and the baseline five-factor model showed negligible variations in the model fit indices (*ΔCFI* = 0.021, *ΔTLI* = 0.011, *ΔRMSEA* = 0.006). These findings further support the conclusion that common method bias does not substantially affect the results of this study.

### 4.2. Descriptive Statistics

Descriptive statistics and intercorrelations among the key variables are summarized in Table 1. As shown, employee–AI collaboration was significantly and positively correlated with job insecurity (*r* = 0.33, *p* < 0.01), and job insecurity was significantly and positively correlated with knowledge hiding (*r* = 0.30, *p* < 0.001). These findings provide preliminary support for the hypothesized relationships in this study.

To clarify the computation of descriptive statistics, the mean values reported in Table 1 for employee–AI collaboration, job insecurity, knowledge hiding, and AI trust represent the average item scores of each construct. Each latent variable was measured using a multi-item scale, and participants’ responses were averaged across all corresponding items to generate a composite score, which is consistent with prior research employing Likert-type measures. Thus, the reported means and standard deviations reflect these composite scale scores rather than latent factor scores estimated through structural equation modeling. In addition, gender was coded as a binary variable (1 = male, 0 = female) for analytical purposes; therefore, its mean represents the proportion of male respondents in the sample.

### 4.3. Confirmatory Factor Analysis

Confirmatory factor analyses (CFA) were performed using Mplus 7.4 to evaluate the distinctiveness of the study constructs. The hypothesized four-factor measurement model was first tested and compared with a series of alternative models, including three-factor, two-factor, and single-factor configurations. The model comparison results, presented in Table 2, show that the proposed four-factor model achieved a substantially superior fit relative to the alternative models (*χ*^2^/*df* = 1.22, *RMSEA* = 0.06, *CFI* = 0.94, *TLI* = 0.95). These findings provide evidence of adequate discriminant validity among the focal constructs and confirm that the overall measurement model appropriately represents the data.

### 4.4. The Results on Reliability and Validity

The results of the reliability and validity tests (Table 3) indicate that all measurement scales used in this study demonstrate satisfactory psychometric properties. First, internal consistency reliability was established across all constructs. The Cronbach’s α values ranged from 0.82 to 0.92, and all composite reliability (CR) values exceeded the recommended threshold of 0.70, with Employee–AI Collaboration (CR = 0.93), AI Trust (CR = 0.91), Job Insecurity (CR = 0.90), and Knowledge Hiding (CR = 0.94), confirming strong reliability.

Second, convergent validity was supported by both factor loadings and the average variance extracted (AVE). As shown in Table 3, all standardized factor loadings were above 0.70, demonstrating adequate indicator reliability. Moreover, the AVE values for all constructs ranged from 0.61 to 0.73, surpassing the recommended minimum of 0.50. These results collectively indicate that each construct explains a substantial proportion of variance in its respective indicators.

In addition to the Fornell–Larcker criterion, discriminant validity was further assessed using the Heterotrait–Monotrait (HTMT) ratio, which is considered a more rigorous indicator of construct distinctiveness. The HTMT values for all construct pairs were below the recommended threshold of 0.85, indicating that the constructs are empirically distinguishable from one another. These results provide additional support for the adequacy of discriminant validity in the measurement model and confirm that each construct captures a unique conceptual domain without excessive overlap.

Finally, the descriptive statistics for the individual indicators revealed reasonable mean values and standard deviations across constructs, suggesting no abnormal response patterns or restricted variance. Overall, the reliability and validity evidence demonstrates that the measurement model is robust and appropriate for subsequent hypothesis testing.

### 4.5. Hypothesis Testing

We employed Mplus 7.4 to test the proposed hypotheses using a series of hierarchical regression models. Specifically, Models 1–4 (M1–M4) correspond to the control-variable model, the main-effect model, the mediation model, and the moderated regression model, respectively. The results of these analyses are summarized in Table 4. 

As shown in M1 in Table 4, employee–AI collaboration was positively associated with job insecurity (*B* = 0.28, *p* < 0.001). Hypothesis 1 was supported. As shown in M4 in Table 4, job insecurity, in turn, showed a significant positive relationship with knowledge hiding (*B* = 0.23, *p* < 0.001). Hypothesis 2 was supported.

To test the mediating role of job insecurity, we applied the conditional indirect effects approach proposed by Preacher and Hayes (2008), using a bootstrap procedure with 5,000 resamples. The indirect effect of employee–AI collaboration on knowledge hiding through job insecurity was 0.06, with a 95% confidence interval of [0.02, 0.08], which excluded zero. These findings indicate that job insecurity significantly mediates the link between collaboration and knowledge hiding. Hypothesis 3 was supported.

**Figure 2:**
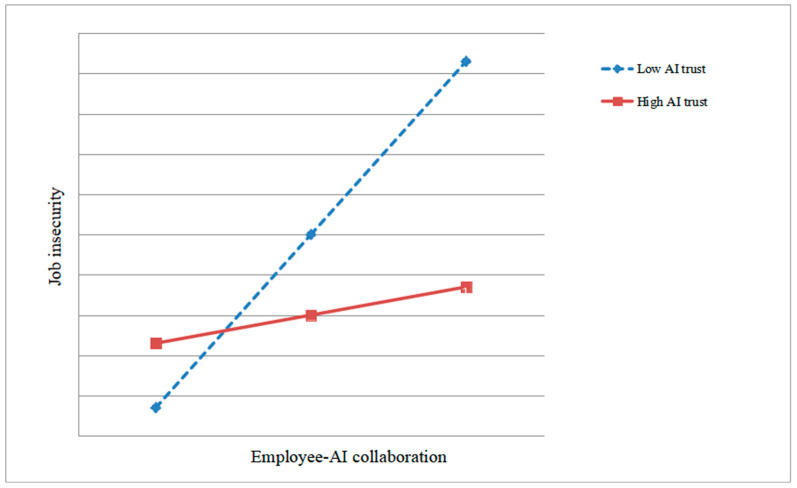
The moderating effect of AI trust.

The results also revealed a significant interaction between employee–AI collaboration and AI trust on job insecurity (*B* = –0.18, *p* < 0.01; see M2 in Table 4). In other words, higher levels of trust in AI weakened the positive association between collaboration and job insecurity. Hypothesis 4 was supported. We plotted the moderating effect of AI trust, as shown in Figure 2.

Finally, the moderated mediation hypothesis was examined using bootstrap estimation with 5000 resamples (Table 5). When AI trust was low, the indirect effect of employee–AI collaboration on knowledge hiding via job insecurity was 0.28 (95% CI [0.12, 0.33]), and the confidence interval did not include zero. Under high AI trust, the indirect effect decreased to 0.13 (95% CI [−0.06, 0.29]), with the confidence interval including zero. The difference between these conditional effects indicates a significant moderated mediation effect. Hypothesis 5 was supported.

## 5. Discussion

First, our findings demonstrate that employee–AI collaboration significantly heightens employees’ perceptions of job insecurity, which is consistent with prior research highlighting AI-induced substitution concerns and skill devaluation (Koo et al., 2021; C. Xu et al., 2024). These studies suggest that employees often interpret AI’s learning speed and automation capabilities as signals that threaten role stability and professional expertise. Extending this line of research, our results show that even when AI is positioned as a collaborative partner rather than a replacement technology, employee–AI collaboration can still be cognitively appraised as a threat to role relevance and future prospects. This finding echoes Chen et al. (2025), who demonstrated that different forms of AI interaction shape employees’ psychological responses. However, unlike studies emphasizing the empowering potential of human–AI interaction—such as enhanced autonomy or capability development (Li et al., 2024)—our results reveal that collaboration itself may function as a persistent source of psychological strain, underscoring the ambivalent nature of employee–AI collaboration.

Second, the positive relationship between job insecurity and knowledge hiding identified in this study aligns with prior research conceptualizing knowledge hiding as a defensive response to perceived threats. Previous studies have shown that employees experiencing insecurity or vulnerability are more likely to withhold knowledge as a self-protective behavior (Serenko & Bontis, 2016; Nguyen et al., 2022; Zhao et al., 2016). Building on this foundation, our findings indicate that when AI becomes an active collaborator in work processes, employees’ threat appraisals extend beyond interpersonal concerns to include worries that their knowledge may be appropriated, replicated, or rendered less distinctive by AI. Such threat-based interpretations intensify employees’ motivation to engage in knowledge hiding as a coping response. In this way, our study deepens existing explanations of knowledge hiding by highlighting a mechanism that is particularly salient in human–AI collaboration contexts.

Finally, the moderating role of AI trust observed in this study provides further insight into employees’ heterogeneous psychological reactions to employee–AI collaboration. Consistent with prior research showing that trust facilitates engagement with AI and reduces perceived risks (Kong et al., 2023; McGrath et al., 2025), our findings demonstrate that AI trust weakens the extent to which employee–AI collaboration is appraised as threatening, thereby buffering job insecurity and its downstream effect on knowledge hiding. This result aligns with studies emphasizing the importance of transparency, reliability, and explainability in building trust and alleviating perceived threats (J. Kim et al., 2021; de Brito Duarte et al., 2023). At the same time, in line with research highlighting skepticism toward AI adoption (Omrani et al., 2022), our findings underscore that trust is not uniform across employees. Rather, AI trust shapes how employees cognitively interpret the meaning of collaboration with AI, offering a clear explanation for why employees working in the same AI-enabled environment may respond in markedly different ways.

### 5.1. Theoretical Contributions

This study contributes to research on human–AI interaction by conceptualizing employee–AI collaboration as an evolving and bidirectional work process rather than as a one-time act of technology adoption or automation. Prior research has largely emphasized AI’s instrumental benefits for efficiency, task optimization, and decision support, often portraying AI as a passive enabler of human work (Ibrahim Hassan et al., 2024; Li et al., 2023). In contrast, this study frames employee–AI collaboration as a form of joint value creation in which humans and AI continuously interact and adapt to one another. From a cognitive appraisal perspective, this interactional view highlights that collaboration with AI can simultaneously be interpreted as opportunity-enhancing and threat-inducing—supporting performance while also prompting concerns about role relevance and professional identity (Li et al., 2025a). By emphasizing employees’ subjective interpretations of collaboration with AI, the study moves beyond the “AI-as-efficiency” narrative and offers a more integrative account of how AI reshapes employees’ work experiences and organizational dynamics.

The study also extends contextual theorizing on knowledge behavior by identifying employee–AI collaboration as a distinct technological context that shapes employees’ knowledge-related decisions. Prior research has primarily examined knowledge hiding through personal traits (e.g., competitiveness, envy), relational dynamics (e.g., trust, leadership style), and organizational climates (e.g., collaborative culture) (Černe et al., 2014; Connelly et al., 2019; Nguyen et al., 2022). Far less attention has been paid to how emerging technologies influence employees’ interpretations of knowledge sharing and withholding. Building on Cognitive Appraisal Theory, our findings suggest that within employee–AI collaboration contexts, perceived substitution threats and concerns about skill devaluation emerge from employees’ threat-based appraisals of their work situation, motivating defensive coping behaviors such as knowledge hiding. In this sense, knowledge hiding reflects employees’ attempts to manage perceived vulnerability and preserve a sense of control and distinctiveness, rather than intentional antisocial behavior. By shifting the analytical focus from interpersonal dynamics to human–AI interaction, this study enriches understanding of how technological transformation reshapes knowledge behavior in organizations.

Finally, by examining AI trust, the study identifies a critical psychological boundary condition that shapes how employees cognitively interpret and respond to AI-enabled work systems. Although trust in technology is a well-established theme in human–AI interaction research, prior studies have focused predominantly on consumers or external users, paying limited attention to intra-organizational contexts (Gerlich, 2024; J. Kim et al., 2021; McGrath et al., 2025; Reinhardt, 2023). Integrating AI trust into a Cognitive Appraisal Theory framework, this study demonstrates that trust influences whether employee–AI collaboration is interpreted as threatening or manageable, thereby shaping employees’ job insecurity and subsequent coping responses. In doing so, the study explains why employees working within the same AI-enabled environment may exhibit markedly different psychological and behavioral reactions (G. Xu & Xue, 2023). This perspective extends trust research beyond interpersonal and leader–employee relationships to human–AI collaboration (Glikson & Woolley, 2020) and highlights the importance of trust-building interventions for fostering adaptive responses and sustained knowledge exchange during technological change.

### 5.2. Practical Implications

First, organizations should redesign their AI implementation processes to directly reduce the job insecurity that employee–AI collaboration may trigger. Instead of merely introducing AI as a technical tool, managers should provide employees with clear, structured information about how AI will affect job roles, task boundaries, and future competency requirements. For example, before deploying AI systems, managers can communicate a task-level analysis that specifies which responsibilities will remain human-led and which will be supported by AI. Involving employees in collaborative work redesign workshops—where they help determine human–AI task division—can further strengthen their sense of control and reduce perceived replacement threats. In addition, organizations should establish ongoing reskilling pathways rather than one-off training, such as multi-stage capability development plans and transparent career progression routes linked to AI-enhanced skills, thereby reinforcing employees’ long-term role security.

Second, to prevent knowledge hiding that arises from heightened insecurity, organizations should implement incentive structures and social mechanisms that reinforce knowledge-sharing norms. Managers can incorporate indicators such as knowledge contribution, cross-functional collaboration, and problem-solving transparency into performance evaluations or team-based rewards. Creating formalized Communities of Practice (CoPs) focused on AI-supported workflows allows employees to exchange practical insights and demonstrate their unique expertise, which helps counteract the perception that their knowledge is easily replaceable by AI. Leaders also play a critical role in shaping a psychologically safe environment: by openly sharing information, acknowledging mistakes, and modeling collaborative behaviors, supervisors signal that knowledge contribution strengthens—not threatens—employees’ standing within the team.

Finally, organizations should adopt targeted strategies to strengthen employees’ trust in AI, as AI trust was found to buffer the insecurity caused by collaboration and reduce downstream knowledge hiding. Enhancing AI transparency and explainability—through demonstrations, “how AI works” briefings, or visual dashboards showing decision logic—can reduce employees’ uncertainty about the system’s reliability and intentions. Managers should also clarify the strategic rationale for AI adoption, emphasizing augmentation rather than substitution, and highlight concrete cases where AI has enhanced employees’ effectiveness. Offering low-risk opportunities for hands-on AI experimentation further builds familiarity and confidence. When employees perceive AI as a dependable and supportive collaborator, they are less likely to interpret AI integration as a threat and, consequently, less inclined to engage in defensive behaviors such as knowledge hiding.

### 5.3. Limitations and Future Research Directions

First, although the three-wave time-lagged design helped mitigate common method bias and improved causal inference, reliance on self-reported data still raises concerns about social desirability and perceptual distortion. In addition, the relatively short intervals between survey waves may have restricted our ability to capture the longer-term effects of employee–AI collaboration on employee outcomes. Future studies could address these limitations by incorporating multiple data sources—such as supervisor evaluations or objective AI usage records—and by employing longitudinal designs that trace the dynamic impact of collaboration over time.

Second, the sample was drawn from knowledge-intensive enterprises in eastern and central China. While this context is well suited to the study’s objectives, it may constrain the generalizability of the findings. Employees’ perceptions of AI, levels of trust, and knowledge behaviors are likely to differ across cultural and institutional environments. For instance, in collectivist settings, individuals may prioritize group goals and suppress defensive behaviors, whereas in more individualist contexts, knowledge hiding may occur more readily. Comparative research across industries and cultures could thus test the boundary conditions and broader applicability of the present conclusions.

Third, our model focused on job insecurity as a mediating mechanism and AI trust as a moderating factor. Yet the psychological and behavioral pathways through which employee–AI collaboration influences outcomes are likely to be more complex. Emotional reactions (e.g., anxiety, stress) and cognitive appraisals (e.g., perceived fairness, sense of control) may also play important roles. Moreover, contextual factors such as leadership style, team climate, or organizational support could shape how employees interpret and respond to AI collaboration. Future research should incorporate these emotional, cognitive, and contextual elements into a more integrative framework.

## 6. Conclusions

In today’s rapidly evolving era of artificial intelligence, collaboration between employees and AI has become a central aspect of organizational functioning. Grounded in Cognitive Appraisal Theory, this study proposed and empirically tested a conceptual framework linking employee–AI collaboration, job insecurity, and knowledge hiding, while considering AI trust as a boundary condition. Employing a three-wave time-lagged research design, several important insights emerged. First, employee–AI collaboration was found to heighten perceptions of job insecurity, reflecting employees’ concerns over the potential threats to job stability and skill relevance. Second, job insecurity positively predicted knowledge-hiding behaviors, suggesting that employees may adopt protective strategies when experiencing uncertainty. Third, job insecurity served as a mediating mechanism through which employee–AI collaboration influenced knowledge hiding, highlighting the underlying psychological process connecting collaboration with knowledge-related outcomes. Finally, AI trust moderated the association between employee–AI collaboration and job insecurity, thereby weakening the indirect impact of collaboration on knowledge hiding.

Theoretically, this study reveals the double-edged nature of employee–AI collaboration, expands contextual explanations of knowledge hiding, and highlights AI trust as a critical boundary condition, thereby extending the application of Cognitive Appraisal Theory to emerging technological contexts. Practically, the findings offer actionable guidance: managers should carefully position human–AI relationships, reduce employees’ insecurity, foster knowledge-sharing environments, and enhance AI trust through transparency, training, and communication. These measures can foster a virtuous cycle between employee–AI collaboration and organizational innovation.

## Figures and Tables

**Table 1:** Means, standard deviations, correlations among study variables.

Variable	Mean	SD	1	2	3	4	5	6	7
1. Gender	-	-	1						
2. Age	32.40	6.80	0.02	1					
3. Employee-AI collaboration	4.21	1.22	0.23	0.11	1				
4. Job insecurity	3.89	1.04	0.32	0.22	0.33 **	1			
5. Knowledge hiding	4.01	0.89	−0.33	0.32	0.27 **	0.30 ***	1		
6. AI trust	2.85	1.23	0.22	0.10	0.24 **	−0.22 **	−0.31 **	1	

Note: ** *p* < 0.01, *** *p* < 0.001, *SD* = standard deviation.

**Table 2:** Confirmatory Factor Analysis.

Model	*χ*^2^/*df*	*CFI*	*TLI*	*RMSEA*
Four-factor model	1.22	0.94	0.95	0.06
Three-factor model	6.34	0.74	0.70	0.15
Two-factor model	8.55	0.48	0.44	0.21
One-factor model	10.64	0.42	0.39	0.34

Notes: CFI = Comparative Fit Index; *TLI* = Tucker–Lewis Index; *RMSEA* = Root Mean Square Error of Approximation.

**Table 3:** Reliability and Validity for Each Variables.

Variables	Items	Mean	*SD*	Factor Loading	AVE	CR
Employee-AI collaboration	A1	4.25	1.21	0.83	0.73	0.93
	A2	4.18	1.20	0.86		
	A3	4.30	1.23	0.88		
	A4	4.15	1.19	0.84		
	A5	4.17	1.24	0.87		
AI trust	B1	2.90	1.25	0.78	0.65	0.91
	B2	2.83	1.22	0.80		
	B3	2.88	1.2	0.82		
	B4	2.79	1.26	0.84		
	B5	2.91	1.23	0.79		
	B6	2.87	1.21	0.83		
Job insecurity	C1	3.92	1.03	0.79	0.62	0.90
	C2	3.88	1.04	0.81		
	C3	3.91	1.02	0.78		
	C4	3.86	1.05	0.77		
	C5	3.85	1.04	0.80		
	C6	3.90	1.06	0.82		
	C7	3.93	1.03	0.84		
Knowledge hiding	D1	4.02	0.92	0.74	0.61	0.94
	D2	4.01	0.91	0.77		
	D3	3.98	0.9	0.79		
	D4	4.05	0.88	0.81		
	D5	4.03	0.87	0.76		
	D6	4.00	0.89	0.78		
	D7	4.07	0.90	0.82		
	D8	4.09	0.91	0.80		
	D9	3.96	0.92	0.75		
	D10	4.10	0.88	0.83		
	D11	3.97	0.93	0.81		
	D12	4.00	0.89	0.79		

Notes: AVE = Average Variance Extracted; CR = Composite Reliability; *SD* = Standard Deviation.

**Table 4:** Results of Regression Analysis.

Model	Job Insecurity				Knowledge Hiding			
	M1		M2		M3		M4	
	*B*	*SE*	*B*	*SE*	*B*	*SE*	*B*	*SE*
Gender	0.08	0.06	0.07	0.06	0.05	0.04	0.09	0.05
Age	−0.03	0.04	−0.02	0.04	0.08	0.05	0.08	0.04
Employee-AI collaboration	0.28 ***	0.07	0.25	0.05	0.27 ***	0.06	0.21 ***	0.05
Job insecurity						0.03	0.23 ***	0.04
AI trust			−0.10	0.05				
Int			−0.18 **	0.05				
R^2^	0.06		0.18		0.23		0.34	
F Value	2.1		6.75		7.89		9.34	

Note: ** *p* < 0.01, *** *p* < 0.001, B = unstandardized regression coefficient; *SE* = standard error; Int = interaction term (Employee–AI collaboration × AI trust); *R*^2^ = coefficient of determination.

**Table 5:** Moderated Mediation Effect Test Results.

Moderator	Effect	Standard Error	Lower Limit of 95% Confidence Interval	Higher Limit of 95% Confidence Interval
Mean − 1 SD	0.28	0.03	0.12	0.33
Mean + 1 SD	0.13	0.27	–0.06	0.29

## Data Availability

The raw data supporting the conclusions of this article will be made available by the authors on request.
